# 2353. SARS-CoV-2 Variants among US Veterans after COVID-19 Bivalent Vaccine Administration

**DOI:** 10.1093/ofid/ofad500.1974

**Published:** 2023-11-27

**Authors:** Mark Holodniy, Ying Pei, Gary Stack, Christopher L Wade, Yashpal Agrawal, Nicholas Barasch, Fady Baddoura, M Carmen Frias-Kletecka, J Stacey Klutts, Jessica Wang-rodriguez

**Affiliations:** Department of Veterans Affairs, Palo Alto, California; Boise VA Medical Center, Boise, Idaho; VA Connecticut Health Care System, West Haven, Connecticut; Richard L. Roudebush VA Medical Center, Indianapolis, Indiana; Eastern Colorado VA Health Care System, Denver, Colorado; James J. Peters VAMC/Columbia University Medical Center, New York, New York; Orlando VA Medical Center, Orlando, Florida; US Department of Veterans Affairs, Los Angeles, California; Iowa City VA Health Care System, Iowa City, IA; Department of Veteran's Affairs, Washington, DC, District of Columbia

## Abstract

**Background:**

The US Department of Veterans Affairs (VA) created the Sequencing For Research, Clinical, Epidemiology (SEQFORCE) Program in July 2021 to conduct SARS-CoV-2 Whole Genome Sequencing (WGS). Herein, we describe SARS-CoV-2 variants associated with COVID-19 infection, including after bivalent vaccination.

**Methods:**

Demographics, COVID-19 vaccinations, hospitalizations, SARS-CoV-2 variants, and Charlson comorbidity index variables were extracted from VA data sources from 7/1/21-4/1/23. Eligible respiratory samples required a positive RT-PCR result from any platform with cycle threshold < 30. WGS was performed using 3 different platforms (Clear Labs, Illumina, ThermoFisher) at the 9 laboratories and one analytic pipeline (PraediGene, Bitscopic) using Pangolin and Nextclade. Post-vaccine COVID-19 infection was defined as > 2 weeks after COVID-19 vaccine receipt.

**Results:**

Over 41,000 samples from 150 VA clinical sites across all geographical locations in the country have been analyzed by 9 SEQFORCE laboratories since July 1, 2021 (Figure 1), including 28,800 after vaccine breakthrough infection (Table 1). Since October 1, 2022, 3,087 patients had SARS-CoV-2 variants characterized including 986 after bivalent vaccination, 1,191 who were fully vaccinated (including Janssen), but not boosted and didn’t receive a bivalent vaccine dose, and 910 who were never vaccinated (Figure 2) according to VA records. Those who received bivalent vaccine were significantly older, male, and had higher Charlson morbidity scores compared to those not receiving a bivalent vaccine. There was no difference in infection rates based on type of bivalent vaccine received. Bivalent vaccine recipients had significantly more XBB and less BA.5 variants, compared to not receiving a bivalent vaccine (p < 0.0001 for both comparisons).

**Figure:**
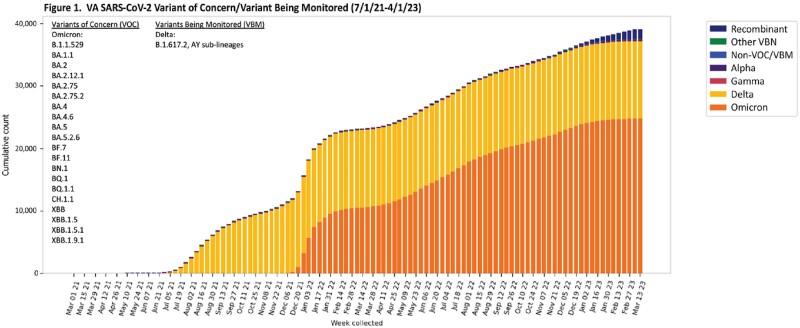

**Figure:**
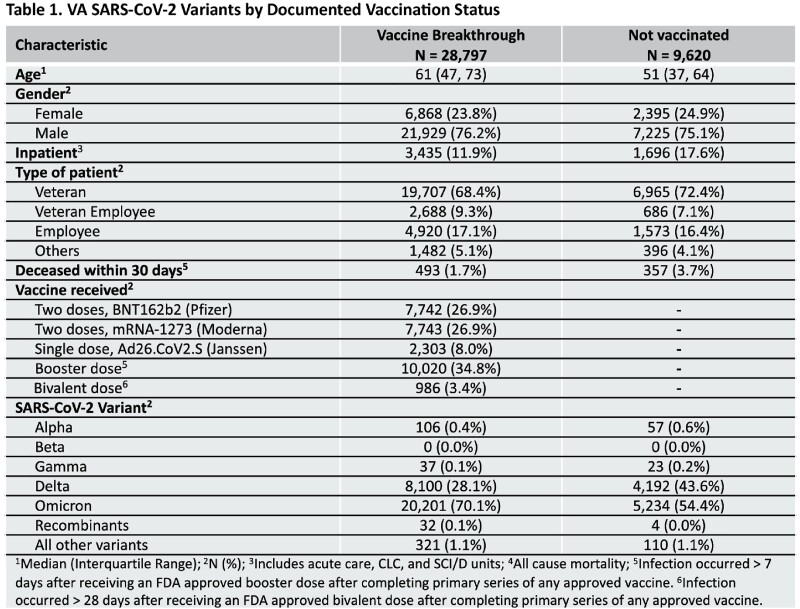

**Figure:**
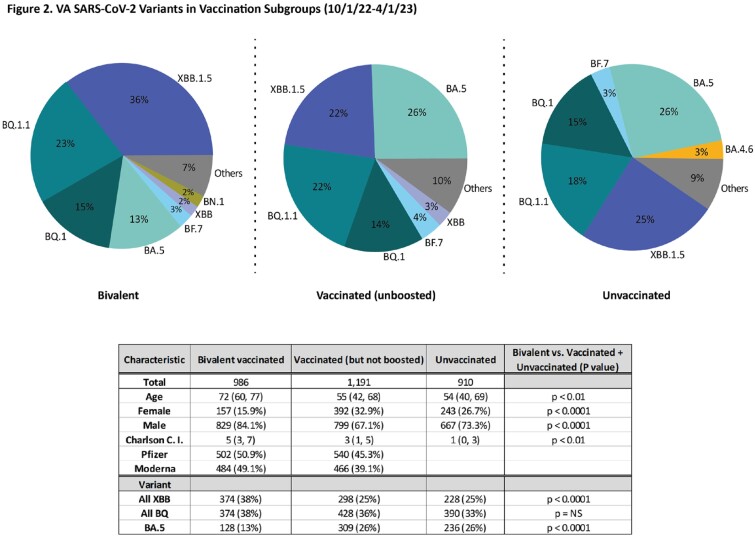

**Conclusion:**

VA established a SARS-CoV-2 sequencing consortium to track variants for clinical and epidemiological indications. Sample submission was voluntary and therefore may have limited geographic, temporal and clinical diversity among patient samples analyzed. Significantly more XBB and less BA.5 variants were found after bivalent vaccination infection compared to other contemporaneous variants among those not receiving bivalent vaccine.

**Disclosures:**

**M. Carmen Frias-Kletecka, MD**, Sanofi: Honoraria

